# Survival is Better After Breast Conserving Therapy than Mastectomy for Early Stage Breast Cancer: A Registry-Based Follow-up Study of Norwegian Women Primary Operated Between 1998 and 2008

**DOI:** 10.1245/s10434-015-4441-3

**Published:** 2015-03-06

**Authors:** Olaf Johan Hartmann-Johnsen, Rolf Kåresen, Ellen Schlichting, Jan F. Nygård

**Affiliations:** 1 Cancer Registry of Norway, Oslo, Norway; 2 University of Oslo, Oslo, Norway; 3 Nesøya, Norway; 4 Department of Breast and Endocrine Surgery, Oslo University Hospital, Oslo, Norway

## Abstract

**Background:**

Breast-conserving therapy (BCT) and mastectomy (MTX) has been considered to have a similar long-time survival. However, better survival in women undergoing BCT compared with MTX is found in two recent register studies from the United States. The purpose of this study was to compare survival after BCT and MTX for women with early-stage breast cancer in Norway.

**Methods:**

Women with invasive, early-stage breast cancer (1998–2008) where BCT and MTX were considered as equally beneficial treatments were included for a total of 13,015 women. Surgery was divided in two main cohorts (primary BCT, primary MTX) and five subcohorts. Analyses were stratified into T1N0M0, T2N0M0, T1N1M0, T2N1M0, and age groups (<50, 50–69, ≥70). Overall survival and breast cancer-specific survival (BCSS) were calculated in life tables, hazard ratios by Cox regression, and sensitivity analyses.

**Results:**

Five-year BCSS for women who underwent primary BCT or primary MTX was 97 and 88 %, respectively. Women who underwent primary MTX had a hazard ratio of 1.64 (95 % confidence interval 1.43–1.88) for breast cancer death compared with women who underwent primary BCT after adjusting for the year of diagnosis, age at diagnosis, stage, histology, and grade.

**Conclusions:**

Survival was better or equal after breast-conserving therapy than mastectomy in all early stages, surgical subcohorts, and age groups. This advantage could not only be attributed to differences in tumor biology.

## Introduction

The clinical trials comparing breast-conserving therapy (BCT) with mastectomy (MTX) were done decades ago.^1^–^6^ Since these studies were conducted, treatment and especially adjuvant therapy have changed and survival improved.^7^,^8^

In 2013, Hwang et al. published a paper reporting better survival among patients undergoing BCT compared with MTX, challenging the notion of equality in survival between BCT and MTX.^9^ They suggested that differences in tumor biology might have contributed to survival differences between BCT and MTX. In January 2014, Agarwal et al. published a paper corroborating the results of Hwang.^10^ They assumed that a difference in the breast cancer-specific survival rate between BCT and MTX might be due to differences in compliance to adjuvant therapy or tumor biology. Because these two studies were not randomized trials but observational studies, more studies on this topic are needed, especially outside the United States.

In Norway, all cancer cases have to be reported to the Cancer Registry of Norway, making this a complete register for the whole population of Norway with the possibility to form a cohort where, according to national guidelines, BCT and MTX are considered as equal treatment options.^11^ The purpose of this study was to compare differences in survival after BCT and MTX for women with early-stage breast cancer in Norway.

## Materials and Methods

In this study, data from the Cancer Registry of Norway containing information on diagnosis, time of diagnosis, surgery type, surgery month, morphology, tumor grade, and TNM classification (done according to Union of International Cancer Control) were used.^12^

### Cohort Selection

A total of 27,182 female residents of Norway were diagnosed with invasive, primary, early-stage breast cancer during the period January 1, 1998 to December 31, 2008. In this study, early-stage breast cancer is defined as T1–2 N0–1 M0 and stratified into T1N0M0, T2N0M0, T1N1M0, and T2N1M0 (tumor size ≤ 5 cm and 0–3 ipsilateral axillary nodes with metastasis). From these women, a cohort who, according to the Norwegian Breast Cancer Group (NBCG) recommendations, could be offered either MTX or BCT was selected.^7^

The women excluded were as follows: women with previous cancer (2501), women diagnosed with more than one primary breast cancer in same or contralateral breast within 3 months (840), women who did not undergo surgery or information about the operation was missing (2153 of these 41 % were aged ≥ 80 years), missing information about metastasis status (4919 of these 35 % underwent BCT as primary and 65 % underwent MTX as primary), unknown size of tumor or unknown nodal status (2196), final BCT (BCT operated once and BCT with reoperation) not received or missing information on RT (1073), final BCT receiving RT more than 365 days after diagnosis (62), women who received radiotherapy after MTX when nodal axillary status was negative (399), and women who died within 3 months after primary operation (24). The final cohort consists of 13,015 women.

### Surgical Cohorts

Surgery was divided into two main cohorts: primary BCT and primary MTX. Primary BCT was further divided into three subcohorts: BCT operated once, BCT with reoperation, and BCT followed by MTX. Primary MTX was divided into two subcohorts: MTX operated once and MTX with reoperation. Division of surgical main and subcohorts was done 3 months after primary operation. If no further operation was done 3 months after primary operation, the operation was defined as one operation (BCT operated once and MTX operated once). If the women underwent two or more surgeries within 3 months after primary the operation, the operation was defined as several (BCT with reoperation, BCT followed by MTX, and MTX with reoperation).

### Treatment Recommendations from the Norwegian Breast Cancer Group Between 1998 and 2008

NBCG criteria to accept BCT as final result of surgery was as follows: free margin should be at least 5 mm from 1998 to 2003 and 3 mm from 2003 to 2008; an acceptable cosmetic result obtained; tumor size < 5 cm from 2003; multifocal tumors were not accepted from 1998 to 2003; multifocal tumors < 1 cm apart were accepted for BCT from 2003.

Radiation therapy: all women undergoing BCT as final treatment should receive RT. Women younger than 55 years undergoing MTX with one to three positive nodes in axilla were recommended RT to chest wall and axilla from year 1998 to 2003; the age was increased to 70 years from 2003. Women undergoing MTX also were recommended RT if margins were not free. Radiation therapy was deemed given if the patient received a total dose of 47 Gy or more and start of treatment was no more than 365 days from date of diagnosis.

Neoadjuvant treatment is not recommended for early-stage breast cancer. Furthermore, choice of surgery did not influence recommendations of adjuvant chemotherapy or antiestrogen therapy.

### Statistical Analyses

Life tables for 5-year overall survival (OS) and breast cancer-specific survival (BCSS) were stratified by primary BCT, primary MTX, and the following age groups: <50 years, 50–69 years, and ≥70 years. Furthermore, the surgical main cohorts were stratified in grade 1–3, ductal carcinoma, T1N0M0, T2N0M0, T1N1M0, T2N1M0, age < 50, age 50–69, and age ≥ 70 years. Kaplan–Meier curves were stratified in T1N1M0, grade 3, ductal carcinoma, and age 50–69 years in the surgical main and sub cohorts.

Cox proportional hazards were performed to estimate crude and adjusted hazard ratios for OS and BCSS between BCT and MTX in the surgical main and subcohorts. Cox analyses were performed in the following strata: surgical main cohorts; surgical sub cohorts; first 3 and last 3 years of the study period; women aged < 50 years; women aged 50–69 years; women aged ≥ 70 years; T1N0M0 grade1; T1-2N1M0 where primary MTX received RT and T1-2N1M0 where primary MTX did not receive RT. Furthermore, multivariate analysis was performed were all women receiving RT after MTX were excluded from the cohort. The multivariate analysis was adjusted in the surgical subcohorts for the year of diagnosis, stage, age, histology, and grade. Deterministic sensitivity analyses were performed on misclassification of surgery, selection bias, and uncontrolled confounding according to Greenland.^13^ Statistical analyses were conducted in STATA version 13.1 (StataCorp, Texas, USA).

## Results

Of the 13,015 women with early-stage breast cancer, 8065 (62 %) underwent primary BCT and 4950 (38 %) underwent primary MTX. Table 1 shows clinical characteristics of the patient cohort.

**Table 1:** Baseline characteristics

	Surgical main cohorts				Surgical sub cohorts				
	Number 13,015		Reoperated^a^		Number 13,015				
	PrimaryBCT	PrimaryMTX	BCT	MTX	BCTonce	BCTreop.	BCT-MTX	MTX once	MTX reop.
Number of patients	8065	4950	1481	164	6583	1287	194	4786	164
Proportion of patients	62	38			50.6	9.9	1.5	36.7	1.3
Median follow-up time	7.3 years	7.0 years			7.3 years	7.5 years	8.7 years	7.0 years	7.0 years
Proportion RT	99.3	30.7			100	100	70.1	30.3	43.3

Year of diagnosis	Annual proportion (100 %)		Reoperated^a^		Annual proportion (100 %)				
			BCT	MTX					
1998	34 % (264)	66 % (512)	16 %	3.3 %	29 %	3.1 %	2.2 %	63.8 %	2.2 %
1999	35 % (279)	65 % (520)	18 %	6.3 %	29 %	3.9 %	2.4 %	61.0 %	4.1 %
2000	41 % (364)	59 % (519)	14 %	3.5 %	35 %	4.0 %	1.8 %	56.7 %	2.0 %
2001	53 % (482)	47 % (422)	21 %	4.3 %	42 %	9.2 %	1.9 %	44.7 %	2.0 %
2002	63 % (736)	37 % (436)	23 %	3.9 %	49 %	11.9 %	2.2 %	35.8 %	1.5 %
2003	72 % (967)	28 % (384)	23 %	3.6 %	55 %	14.9 %	1.6 %	27.4 %	1.0 %
2004	73 % (1005)	26 % (368)	20 %	2.2 %	58 %	13.9 %	1.0 %	26.2 %	0.6 %
2005	72 % (1042)	28 % (407)	17 %	2.7 %	60 %	10.8 %	1.2 %	27.3 %	0.8 %
2006	71 % (962)	29 % (388)	18 %	2.8 %	59 %	11.0 %	1.6 %	27.9 %	0.8 %
2007	68 % (990)	32 % (465)	17 %	1.3 %	57 %	10.5 %	0.9 %	31.5 %	0.4 %
2008	65 % (974)	35 % (529)	14 %	2.1 %	56 %	8.3 %	0.9 %	34.5 %	0.7 %
Age at diagnosis (years)									
<30	61 % (31)	39 % (20)	26 %	5.0 %	45 %	16 %	0 %	37 %	2.0 %
30–39	56 % (287)	44 % (222)	26 %	5.0 %	42 %	11 %	3.9 %	41 %	2.2 %
40–49	66 % (1467)	34 % (761)	20 %	4.5 %	53 %	11 %	2.1 %	33 %	1.5 %
50–59	72 % (3024)	29 % (1203)	19 %	3.7 %	58 %	12 %	1.5 %	27 %	1.0 %
60–69	72 % (2515)	29 % (1024)	17 %	2.5 %	59 %	11 %	1.1 %	28 %	0.7 %
70–79	38 % (651)	62 % (1062)	16 %	2.9 %	32 %	5 %	1.3 %	60 %	1.8 %
≥80	12 % (90)	88 % (658)	13 %	2.6 %	10 %	1 %	0.5 %	86 %	2.3 %
TNM stage									
T1N0	75 % (5165)	25 % (1686)	17 %	2.4 %	63 %	11 %	1.7 %	24 %	0.6 %
T2N0	44 % (888)	56 % (1140)	20 %	2.4 %	35 %	8 %	1.2 %	55 %	1.3 %
T1N1	60 % (1340)	40 % (893)	22 %	4.4 %	47 %	11 %	2.0 %	38 %	1.7 %
T2N1	35 % (672)	65 % (1231)	20 %	4.6 %	28 %	7 %	0.5 %	62 %	3.0 %
Histology									
Ductal c.	62 % (6618)	38 % (3981)	18 %	3.5 %	51 %	10 %	1.5 %	36 %	1.3 %
Lobular c.	58 % (756)	42 % (549)	24 %	2.6 %	44 %	12 %	2.0 %	41 %	1.1 %
Other c.	62 % (691)	38 % (420)	19 %	2.6 %	51 %	10 %	1.3 %	37 %	1.0 %
Grade									
I	73 % (2261)	27 % (844)	16 %	2.7 %	61 %	11 %	1.1 %	26 %	0.7 %
II	61 % (3600)	39 % (2259)	17 %	3.3 %	51 %	9 %	1.3 %	37 %	1.3 %
III	54 % (1650)	46 % (1382)	23 %	4.1 %	42 %	10 %	2.3 %	44 %	1.8 %
Unknown	54 % (554)	46 % (465)	22 %	2.4 %	42 %	10 %	1.7 %	45 %	1.1 %

*BCT once* BCT operated once, *BCT reop.* BCT followed by reoperation, *BCT*-*MTX* BCT followed by MTX, *MTX once* MTX operated once, *MTX reop.* MTX with reoperation

^a^BCT reoperated is calculated by number of primary BCT undergoing BCT reoperation and BCT followed by MTX. MTX reoperated is calculated by number of primary MTX undergoing MTX with reoperation

RT was given to 99.3 % in primary BCT and 30.7 % in primary MTX. In the subcohorts, RT was given to 100 % in BCT operated once, 100 % in BCT with reoperation, 70 % in BCT followed by MTX, 30 % in MTX operated once, and 43 % in MTX with reoperation. The proportion of women who underwent primary BCT is highest among women aged 50–69 years. Of women aged 70–79 years, 62 % were operated with primary MTX. At age 80 years and older, 88 % were operated with primary MTX.

### Impact of Surgery Type on Overall and Breast Cancer-Specific Survival

A total of 2,475 deaths were identified in the cohort during the study period, including 1,132 (1,083 after 10 years) due to breast cancer. The 5-year OS was 89 %, and BCSS was 94 % (Table 2). Life tables showed better survival for women undergoing BCT compared with MTX. For women who underwent primary BCT or primary MTX, the 5-year BCSS was 97 and 88 %, respectively. In the age group 50–69 years, the 5-year BCSS for those who underwent primary BCT was 98 and 90 %, respectively.

**Table 2:** Survival by surgical main and subcohorts

	Median age	Total number at start	Number of overall deaths in 5-year period	Overall survival	Number of breast cancer deaths	Breast cancer survival
Survival						
5-year	59.0	13,015	1334	89 %	742	94 %
10-year	59.0	9814	2260	78 %	1083	89 %
Surgical main cohorts						
5-year survival BCT	56.9	8065	412	95 %	225	97 %
5-year survival MTX	62.4	4950	922	80 %	517	88 %
10-year survival BCT	56.9	6370	796	86 %	384	93 %
10-year survival MTX	62.4	3444	1464	64 %	699	82 %
Age < 50 years						
5-year survival BCT	43.6	1785	89	95 %	72	96 %
5-year survival MTX	42.7	1003	120	87 %	111	88 %
Age 50–69 years						
5-year survival BCT	58.8	5539	232	95 %	115	98 %
5-year survival MTX	59.0	2227	296	86 %	201	90 %
Age ≥ 70 years						
5-year survival BCT	74.6	741	91	87 %	38	94 %
5-year survival MTX	78.3	1720	506	69 %	205	86 %
Surgical subcohorts, 5-year survival						
BCT operated once	57.0	6583	324	95 %	163	97 %
BCT with reoperation	56.2	1287	67	94 %	48	96 %
BCT followed by MTX	55.2	195	21	89 %	14	92 %
MTX operated once	62.5	4786	884	80 %	484	89 %
MTX with reoperation	59.3	164	38	76 %	33	79 %

**Fig. 1:**
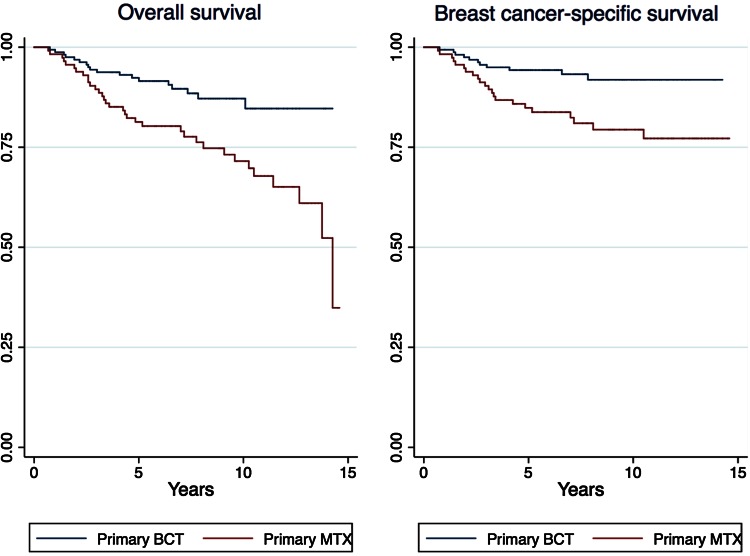
Kaplan–Meier, surgical main cohorts stratified in T1N1M0, grade 3, ductal carcinoma, and age 50–69

The main and surgical subcohorts stratified in stage T1N1M0, grade 3, and ductal carcinoma showed better survival among women undergoing BCT compared with MTX (Kaplan–Meier curves in Fig. 1)

Furthermore, the two main surgical cohorts, primary BCT and primary MTX, were stratified in grade 1–3, ductal carcinoma, and stage (T1N0M0, T2N0M0, T1N1M0, T2N1M0), and none of these strata showed a significant benefit of MTX over BCT; i.e., in all these analyses, BCT was better or equal compared with MTX regarding survival (result not shown in table). Women who underwent MTX with reoperation had the worst prognosis, 79 % 5-year BCSS (Table 2).

In the adjusted Cox analysis, women who underwent primary MTX had a hazard ratio [HR] of 1.64 (95 % confidence interval [CI] 1.43–1.88) for breast cancer death compared with women who underwent primary BCT (Table 3). Adjusted analysis in the beginning of study period (1998–2001) showed HR 1.76 (95 % CI 1.02–3.05) compared with the end of study period (2006–2008) with HR 1.88 (95 % CI 1.23–2.87). Results not shown in table.

**Table 3:** Crude and adjusted HR on overall and breast cancer death in women with early-stage breast cancer

	Crude				Adjusted			
	Overall death		Breast cancer death		Overall death		Breast cancer death	
	HR	95 % CI	HR	95 % CI	HR	95 % CI	HR	95 % CI
Surgical main cohorts								
Primary BCT	1.00	(Reference)	1.00	(Reference)	1.00	(Reference)	1.00	(Reference)
Primary MTX	3.11	(2.86–3.38)	3.16	2.79–3.57	1.65	1.50–1.82	1.64	1.43–1.88
Surgical subcohorts								
BCT once	1.00	(Reference)	1.00	(Reference)	1.00	(Reference)	1.00	(Reference)
BCT reop.	1.05	0.87–1.26	1.38	1.07–1.77	1.04	0.86–1.25	1.28	0.99–1.64
BCT-MTX	1.90	1.39–2.58	2.92	1.97–4.33	1.69	1.24–2.31	2.19	1.47–3.26
MTX once	3.90	2.89–3.47	3.35	2.92–3.85	1.68	1.51–1.86	1.72	1.48–2.01
MTX reop.	4.56	3.60–5.78	7.93	5.94–10.58	2.50	1.96–3.19	3.40	2.53–4.58
Year of diagnosis								
1998	1.00	(Reference)	1.00	(Reference)	1.00	(Reference)	1.00	(Reference)
1999	*0.93*		0.79		*0.89*		0.73	
2000	*0.87*		0.78		*0.9*		0.77	
2001	*0.87*		0.75		*1.05*		*0.83*	
2002	0.69		0.53		*0.89*		0.63	
2003	0.77		0.58		*0.99*		0.76	
2004	0.64		0.45		*0.89*		0.62	
2005	0.69		0.34		*0.87*		0.44	
2006	0.6		0.29		0.75		0.39	
2007	0.59		0.22		0.77		0.30	
2008	0.53		0.17		0.66		0.23	
Age categories (years)								
<30	2.15		3.12		*1.51*		*1.78*	
30–39	1.59		2.42		*1.15*		1.46	
40–49	*0.98*		1.21		0.82		*0.91*	
50–59	1.00	(Reference)	1.00	(Reference)	1.00	(Reference)	1.00	(Reference)
60–69	1.42		*0.96*		1.52		*1.11*	
70–79	3.71		2.15		2.98		1.66	
≥80	8.38		2.92		6.04		2.13	
TNM stage								
T1N0M0	1.00	(Reference)	1.00	(Reference)	1.00	(Reference)	1.00	(Reference)
T2N0M0	2.38		2.95		1.41		1.83	
T1N1M0	1.56		2.86		1.92		2.18	
T2N1M0	3.34		6.57		3.37		3.95	
Histology								
Ductal c.	1.00	(Reference)	1.00	(Reference)	1.00	(Reference)	1.00	(Reference)
Lobular c.	*1.04*		*0.88*		*0.93*		*0.91*	
Other	*1.03*		*0.89*		*1.04*		*1.02*	
Grade								
I	1.00	(Reference)	1.00	(Reference)	1.00	(Reference)	1.00	(Reference)
II	1.69		2.61		1.41		1.98	
III	2.47		5.65		1.92		3.53	
Unknown	1.73		2.84		1.30		1.99	

Numbers in italic are not significant (*p* > 0.05)

Women younger than 50 years who underwent primary MTX had HR 1.58 (95 % CI 1.22–2.04) for breast cancer death compared with women who underwent primary BCT (Table 4). A stratified adjusted analysis performed for women aged 50–69 years (screening age) who underwent primary MTX showed an HR of 1.64 (95 % CI 1.35–1.99) for breast cancer death compared with women who underwent primary BCT with base HR 1.00.

**Table 4:** Crude and adjusted HR on overall and breast cancer death in women with early-stage breast cancer stratified in women <50 years, women aged 50–69 years, women aged ≥70 years, and T1N0M0 grade 1

		Crude		Adjusted	
		Overall death	Breast cancer death	Overall death	Breast cancer death
		HR	HR	HR	HR
*Women aged < 50 years*					
Surgery	Nr				
Primary BCT	1785	1.00 (Reference)	1.00 (Reference)	1.00 (Reference)	1.00 (Reference)
Primary MTX	1003	2.10 (1.71–2.59)	2.51 (1.98–3.18)	1.43 (1.15–1.80)	1.58 (1.22–2.04)
Surgical subcohorts					
BCT once	1415	1.00 (Reference)	1.00 (Reference)	1.00 (Reference)	1.00 (Reference)
BCT reop.	304	0.88 (0.57–1.35)	0.84 (0.50–1.41)	0.85 (0.54–1.31)	*0.80 (0.48*–*1.35)*
BCT-MTX	66	1.51 (0.79–2.87)	1.50 (0.70–3.23)	1.45 (0.75–2.79)	1.32 (0.60–2.87)
MTX once	957	1.96 (1.56–2.46)	2.32 (1.79–3.01)	1.35 (1.06–1.72)	1.46 (1.11–1.93)
MTX reop.	46	5.42 (3.42–8.61)	6.41 (3.87–10.62)	2.95 (1.83–4.75)	3.12 (1.86–5.25)
*Women aged 50–69 years*					
Surgery type					
Primary BCT	5539	1.00 (Reference)	1.00 (Reference)	1.00 (Reference)	1.00 (Reference)
Primary MTX	2227	2.40 (2.12–2.72)	3.26 (2.74–3.88)	1.74 (1.52–2.00)	1.64 (1.35–1.99)
Surgical subcohorts					
BCT once	4546	1.00 (Reference)	1.00 (Reference)	1.00 (Reference)	1.00 (Reference)
BCT reop.	891	1.14 (0.90–1.43)	1.83 (1.34–2.49)	1.09 (0.87–1.39)	1.71 (1.25–2.33)
BCT-MTX	102	2.24 (1.47–3.40)	3.95 (2.32–6.71)	2.03 (1.33–3.09)	2.91 (1.70–6.28)
MTX once	2157	2.42 (2.13–2.78)	3.65 (3.99–4.45)	1.77 (1.35–2.05)	1.85 (1.49–2.30)
MTX reop.	70	5.35 (3.77–7.59)	11.70 (7.77–17.62)	3.32 (2.31–4.78)	4.09 (2.66–6.28)
*Women aged ≥ 70 years*					
Surgery type					
Primary BCT	741	1.00 (Reference)	1.00 (Reference)	1.00 (Reference)	1.00 (Reference)
Primary MTX	1720	2.15 (1.84–2.51)	2.23 (1.67–2.96)	1.56 (1.31–1.85)	1.50 (1.10–2.05)
Surgical subcohorts					
BCT once	622	1.00 (Reference)	1.00 (Reference)	1.00 (Reference)	1.00 (Reference)
BCT reop.	92	*1.16 (0.77*–*1.76)*	*1.05 (0.47*–*2.32)*	*1.01 (0.72*–*1.66)*	*0.83 (0.34*–*1.84)*
BCT MTX	27	*1.42 (0.75*–*2.69)*	*2.68 (1.06*–*6.76)*	*1.30 (0.68*–*2.47)*	*1.92 (0.75*–*4.90)*
MTX once	1672	2.25 (1.89–2.67)	2.36 (1.72–3.23)	1.61 (1.33–1.93)	1.52 (1.08–2.13)
MTX reop.	48	1.67 (1.06–2.64)	2.82 (1.43–5.62)	*1.36 (0.85*–*2.16)*	2.14 (1.06–4.31)
*T1N0M0 grade 1*					
Surgery type					
Primary BCT	1451	1.00 (Reference)	1.00 (Reference)	1.00 (Reference)	1.00 (Reference)
Primary MTX	366	2.61 (1.91–3.56)	3.52 (1.80–6.90)	1.77 (1.22–2.56)	2.07 (0.94–4.56)
Surgical sub cohorts					
BCT once	1245	1.00 (Reference)	1.00 (Reference)	1.00 (Reference)	1.00 (Reference)
MTX once	358	2.61 (1.88–3.61)	4.73 (2.22–10.10)	1.80 (1.23–2.63)	2.80 (1.18–6.61)

In the T1N0M0 grade 1 strata, 17 primary BCT died and 18 primary MTX died

Numbers in italics are not significant (*p* > 0.05)

Women not recommended to receive chemotherapy or antiestrogen therapy, i.e., T1N0M0 grade 1, who underwent primary MTX had an HR of 2.07 (95 % CI 0.94–6.61) compared with women who underwent primary BCT.

Women with node-positive disease (T1-2N1M0) where all in the primary MTX strata received RT gave a primary MTX HR of 2.13 (95 % CI 1.52–2.98) compared with primary BCT with base HR 1.00 (result not shown in table). Respectively, women with node-positive disease (T1-2N1M0) where no one in the primary MTX received RT gave a primary MTX HR of 2.16 (95 % CI 1.78–2.61) compared with primary BCT with base HR 1.00 (result not shown in table). MTX stage T1-2N1M0 shows no advantage of receiving RT. Multivariate analysis where all women receiving RT after MTX were excluded (1,521 women) gave a primary MTX HR of 1.51 (95 % CI 1.27–1.80) compared with primary BCT with base HR 1.00 (result not shown in table).

Women operated with BCT followed by MTX were found to have a worse prognosis compared with women who underwent BCT operated once, BCT with reoperation, and MTX operated once in the adjusted analysis. Nevertheless, only 0.7 % of the primary BCT cohort underwent BCT followed by MTX without receiving RT.

After MTX, 164 underwent reoperation. This group had the worst prognosis in the cohort; 34 % (55) died of breast cancer during a median follow-up time of 6.9 years.

### Sensitivity Analyses

When assuming a dichotomous unmeasured confounder to present in 20, 40, 60, and 90 % of the women undergoing MTX, and 10 % in among women undergoing BCT, the rate ratios were of 2.57, 1.78, 1.36, and 1.01, respectively. In these analyses, we assumed the relative risk of confounding to be 5. Sensitivity analyses of misclassification of surgery and selection bias did not show lower risk between MTX and BCT than in the crude analysis.

## Discussion

The main finding of this study is that both OS and BCSS were better in women with early-stage breast cancer undergoing BCT compared with MTX. This is contrary to the general consensus that MTX and BCT patients have a similar long-time survival, but corresponds well with the two studies done in the United States by Whang and Agarwal, who found better survival in women undergoing BCT compared with MTX.^1^–^3^,^5^,^10^,^14^–^16^

### Possible Selection Effects

The present study is an observational study, and several possible selection effects might explain the observed differences; i.e., the observed differences might be due to other than the surgical procedures. In the following, we discuss the most probable selection effects that might have influenced the observed results.

### Completeness

The Cancer Registry of Norway during the period 2001–2005 had an overall completeness on cancer estimated at 98.8 %.^11^ Selection bias due to missing registration is thus unlikely. There was a higher proportion of patients undergoing primary MTX without known distant metastasis status than primary BCT, 65 versus 35 %, before cohort selection. Nevertheless, information on distant metastasis status was available for all patients in the analyzed cohorts; i.e., they were metastasis-free at the time of diagnosis (M0).

### Access to Health Care

Almost every inhabitant in Norway receives the same health care offer regardless of private insurance, and only public hospitals provide treatment of breast cancer. This might be in contrast to the United States, where women with a higher socioeconomic status are more likely to undergo BCT.^17^,^18^

### Comorbidity

Some of the women underwent MTX due to an overall judgment of their health situation. We have no information on comorbidity; however, the difference between OS and BCSS in women younger than aged 50 years was 1 % in both the primary BCT and primary MTX strata, 3 % for women undergoing BCT, and 4 % for women undergoing MTX at age 50–69 years. This indicates that there are small differences in serious comorbidity in women younger than age 70 years between the two cohorts. However, comorbidity has probably influenced the choice of MTX among the older women.

### Hereditary Breast Cancer

We are not able to stratify for women with hereditary breast cancer, because BRCA1/2 or prophylactic MTX is not recorded in the Cancer Registry. However, in a population-based incidence study in one of the counties in Norway, it was shown that 2.5 % of the women studied were mutation carriers.^19^ This fraction might have a slight detrimental effect on survival in the MTX cohort.

### Patients Own Choice

In a hospital in Norway, 14 % of the women operated for breast cancer underwent MTX because of the patient’s own request, or the cancer had preoperatively been considered more prevalent than at the final histological examination.^20^ This might seem like a low proportion. However, a study from the United States regarding involvement in decision making about surgery for early-stage breast cancer showed that 9 % underwent primary MTX based on patient preference.^21^

### Tumor Biology

When surgery is decided, results from cytology or biopsy together with mammogram and ultrasound normally give information on morphology, grade, and tumor size. Details on tumor biology, such as lymph vascular invasion, are normally not known when the decision on type of surgery is made, and therefore do not explain the difference between BCT and MTX. Furthermore, routine examination on HER2 was recommended from June 2005, late in the study period; therefore, triple-negative disease cannot explain the difference in survival between BCT and MTX.

### Radiation Therapy

Today’s guidelines from NBCG differ from the guidelines in our study period, and today fewer patients would receive RT based on axillary node positive disease (1–3 lymph nodes). MTX with RT and MTX without RT are not directly comparable in our study, based on different recommendations for RT during the study period, but RT given to women with node-positive disease does not seem to increase the survival benefit of the MTX cohort.

Women undergoing MTX with RT likely represent a high-risk disease. Multivariate analysis where none of the patients in the MTX group received RT showed the benefit of BCT compared with MTX (HR 1.51; 95 % CI 1.27–1.80).

### Adjuvant Therapy

The Cancer Registry is incomplete when it comes to chemotherapy and antiestrogen therapy given. However, recommendations for chemo and antiestrogen therapy are identical for patients undergoing BCT and MTX. In this study it was not possible to see whether women undergoing MTX have less compliance to recommended therapy.

### Sensitivity Analysis of Misclassification, Selection Bias, and Unmeasured Confounder

Sensitivity analyses were done under several different assumptions within the following three areas: misclassification of surgery; selection bias, and unmeasured confounder. However, the larger the difference in the proportion of unmeasured confounding in the two cohorts, the lesser the rate ratio adjusted for unmeasured confounding. In the present study, first when assuming that as much as 90 % of women undergoing MTX had uncontrolled confounding (e.g., compliance to adjuvant therapy), and only 10 % in the BCT cohort, did we find a rate ratio of 1.0. We find it unlikely that the difference in adjuvant therapy was more than 10–30 % between the surgical groups (both surgical groups have the same recommendations to adjuvant therapy), and thus the adjusted rate ratio for unmeasured confounding was found to be 1.78, when assuming 10 and 40 % unmeasured confounding in BCT and MTX, respectively, compared with an unadjusted rate ratio of 3.31.

### Proportion of Women Undergoing BCT Compared with MTX Changed During Study Period

The proportion of BCT at the beginning of study period was lower than at the end of study period, but the benefit of BCT compared with MTX did not seem to change during the study period.

### Strengths and Weaknesses of the Study

The major strength of our study is that the results are based on the whole population of women diagnosed with early-stage breast cancer in Norway during the period January 1, 1998 to December 31, 2008. Dividing the surgical main cohort into five surgical subcohorts made it possible to include women initially treated with BCT followed by MTX without receiving RT. If this had not been done, women initially treated with BCT would have been regarded as BCT without RT and excluded from the cohort.

The weaknesses are that the Cancer Registry lacks information on hormone receptor status and information on given adjuvant therapy. However, neither of these factors determines whether a patient should undergo BCT or MTX.^7^ Observational studies, such as this, are prone to selection effects. However, as discussed above, we find it unlikely that this can explain all of the observed differences in survival among women undergoing BCT compared with MTX.

## Conclusions

This study corroborates the findings of two studies from the United States showing better survival for women undergoing BCT compared with MTX. This advantage could not be attributed to differences in tumor biology. Further studies are necessary to determine whether this benefit is caused by variation in adjuvant therapy or by type of surgery.
